# Barriers to completion of maternal and neonatal continuum of care services in Assosa Zone, north-western Ethiopia

**DOI:** 10.4102/phcfm.v17i1.4718

**Published:** 2025-03-12

**Authors:** Solomon Abtew, Rose M. Mmusi-Phetoe

**Affiliations:** 1 Ethiopian Public Health Association, Addis Ababa, Ethiopia; 2 Department of Public Health, College of Human Sciences, University of South Africa, Pretoria, South Africa

**Keywords:** barriers, continuum of care, maternal and neonatal health, service utilisation, Assosa Zone

## Abstract

**Background:**

The continuum of care (CoC) in maternal and neonatal services among women in Ethiopia was low because of individual and cultural barriers.

**Aim:**

This study aims to identify factors that hindered the utilisation of the CoC services.

**Setting:**

The study took place in the Assosa Zone of north-western Ethiopia.

**Methods:**

A qualitative study using audio-taped individual interviews was conducted. A total of 52 study participants were purposefully recruited from the Assosa Zone. Thematic analysis was employed to identify major themes and categories from the transcripts.

**Results:**

Findings revealed the economic situation of women as the underlying barrier to women accessing and utilising maternal and neonatal CoC services. Presumably, high transport and medical costs and the inability to pay the raised costs were drivers to discontinuity of the CoC of maternal and neonatal services. Other barriers to utilisation of CoC services were found to be workload in the households, secreting pregnancy, traditional beliefs, husbands’ attitude and religion, awareness gaps in pregnancy, and maternal and neonatal care. These factors are thus regarded as important barriers to the utilisation of continuity of care in maternal and neonatal services in Ethiopia.

**Conclusion:**

Moreover, economic, cultural and religious factors, maternal awareness and husbands featured as significant barriers to the utilisation of maternal and neonatal CoC services in Ethiopia.

**Contribution:**

The findings revealed the economic situation of women as a barrier to the CoC in maternal and neonatal services utilisation, manifesting itself in unaffordable transport and medication user fees.

## Introduction

Worldwide, maternal and neonatal deaths continue to be significant public health problems.^1^ The global maternal mortality ratio (MMR) was estimated to be 223 per 1000 live births (lb) in 2020,^2^ while the global neonatal mortality rate (NMR) was 17.3 per 1000 lb in 2022.^3^ The deaths occurred because of pregnancy and birth-related complications. According to a recent report, sub-Saharan Africa alone accounted for 70% of global maternal mortality^4^ and 42% of neonatal mortality.^5^

Ethiopia has been experiencing high rates of maternal^6^ and neonatal^7^ mortality since 2000. Although the MMR declined from 871 per 100 000 lb in 2000 to 267 per 100 000 lb in 2020, Ethiopia remains one of the four countries with the highest burden of maternal deaths, following Nigeria, India and the Democratic Republic of the Congo.^2^ The under-five mortality declined from 123 per 1000 lb in 2005 to 59 per 1000 lb in 2019 in Ethiopia.^8^ However, the country’s NMR remains high, with only a slight decline from 39 deaths per 1000 lb in 2000 to 33 deaths per 1000 lb in 2019.^8^ According to the Ethiopian Demographic Health Survey (EDHS) 2016, neonatal mortality varies across different regions; lowest in Addis Ababa (18 deaths per 1000 lb) and highest in Amhara region (47 deaths per 1000 lb).^9^ The NMR in Benishangul Gumuz Region (BGR) is 35 deaths per 1000 lb, which is higher than the national average.^9^ These disparities in mortality and the presence of different predictors indicate that there is a difference in health services coverage and socio-demographic variations.^9^

The sustainable development goals (SDGs) require nations to lower the global MMR to less than 70 per 100 000 lb^2^ and the NMR to at least 12 per 1000 lb by 2030.^10^ One of the key tactics for achieving SDG 3.1 and SDG 3.2 is the utilisation of a continuum of care (CoC) in maternal and neonatal health (MNH) services.^11^ The CoC has elements of location and time. The time dimension links care delivery across pregnancy, birth and postpartum periods, whereas the location dimension links various levels of healthcare sites such as homes, communities and health institutions. These dimensions emphasise the importance of linkages between care packages and coordination efforts at different levels of a continuum of maternal and neonatal care services.^12^ Maternal and neonatal health CoC services remain underutilised in Ethiopia.^13^ Studies show that Ethiopians only use 12.1% – 37.2% of the continuum of MNH care services available.^14,15,6^ According to the 2016 EDHS, about 93% of women are still not receiving comprehensive MNH CoC services.^17^ In Ethiopia, 72.5% of all deliveries are home-based and most neonatal and maternal fatalities are also home-based.^18^ These occur as a result of multiple barriers, such as personal, community and cultural barriers.^19,20^

Individual and community barriers impede women’s access to health services, affecting their overall health outcomes. These barriers include mistrust of public health facilities, aversion to biomedical interventions, gendered decision-making and restricted mobility.^20^ Culture and religion influence attendance of MNH CoC services from health facilities.^21^ A study in Southern Ethiopia indicates that women believed that people should know about their pregnancy when it is visible to others. This resulted in delaying early antenatal care (ANC) initiation and led to incomplete utilisation of MNH CoC services.^21^ In Ethiopia, delivery at home is considered normal, while women who deliver at health facilities are considered weak.^21^ Muslim followers preferred female health workers for their clinical examinations and assisted them in childbirth.^22^ In Eastern Ethiopia, Muslim religious followers were less likely to seek maternal health services compared to Christian followers.^23^ This is linked to Muslim women believing that their naked bodies could only be seen by their husbands, and accordingly, women preferred female traditional birth attendants to skilled male birth attendants. This belief barred women from receiving MNH CoC services from male health providers.^23^

Maternal and neonatal CoC services are influenced by diverse individual, community and cultural barriers in Ethiopia, as indicated in this section.^16^ The effects of these barriers on the completion of MNH CoC services were not known, and neither were the barriers well studied at the subnational level. Thus, there was a need for further exploration to improve completion of MNH CoC services. Understanding the barriers is crucial in addressing the specific needs of women and neonates and significantly improving the wellness of MNH. Hence, the study aimed to explore the barriers that hinder the utilisation of MNH CoC in Assosa Zone, north-western Ethiopia.

## Research methods

### Study design

A descriptive exploratory qualitative study design was used to discover the deeper views of study participants about the barriers that hinder MNH CoC services. The study was guided by the Andersen Health Behaviour Model^24^ and assumed that the MNH CoC was barred by individual and cultural factors such as lack of awareness, perceptions, fear of complications, previous experiences of ANC, skilled delivery, postnatal care (PNC) services, religious practices, financial constraints and health service-related factors. With the guidance of the Model, the researchers conducted in-depth interviews (IDIs) from January 2022 to March 2022, with study participants gathering data on individual and cultural barriers that hinder the completion of MNH CoC services. The study focussed on understanding the experiences and practices of participants in the study area.

### Study setting

In Ethiopia, the health system is structured into primary, secondary and tertiary levels of care. The primary level of care is found at the district level and comprises primary hospitals, health centres and health posts for the provision of promotive, preventive and curative MNH CoC services. The primary health care provision is linked to the secondary and tertiary through a referral system.

As part of community engagement and empowerment, Ethiopia has expanded community health services through the expansion of the health extension programmes and actively engaged community volunteers to reach most households and communities since 2004.^25^ In 2011, community engagements have restructured and introduced the women Health Development Army (HDA) to strengthen the health extension programme and participation of individuals, families and communities through the support of Kebele (the lowest administrative unit in Ethiopia) leaders.^26^ Under the women’s HDA structure, all women are mobilised in groups to share actionable health messages and influence each other to adapt and practise health behaviours.^27^ Health Development Army leaders support health extension workers (HEWs) in disseminating important messages related to MNH CoC services, identifying pregnant women and linking them to HEWs for early ANC through PNC services. At health facility levels, MNH CoC services such as ANC, skilled delivery and PNC services are provided by health workers. After delivery provision, 24 h of PNC services are provided at the health facility level, and then the consecutive PNC services are provided by HEWs at the home level.

**FIGURE 1:**
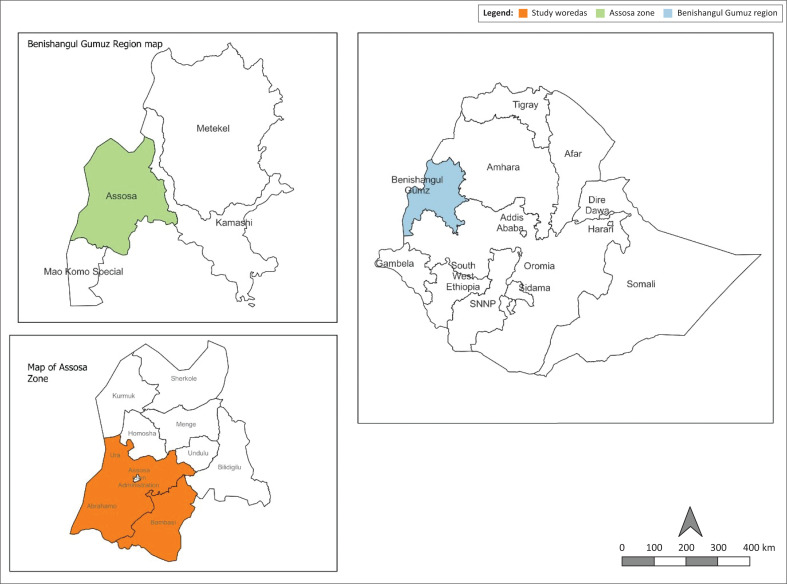
Map showing the study area.

The study was conducted in the Bambasi, Ambrhamo and Ura districts of the Assosa Zone. Assosa Zone is one of the three administrative zones in BGR State and has nine administrative districts. The zone and districts were selected purposively based on the focus of the researchers to get deep insights into barriers that hindered utilisation of MNH CoC services (Figure 1).

### Study population and sample size

Five different groups of participants were selected for the face-to-face interviews. These consisted of women who had given birth in the past 9 months prior to the data collection, community leaders (Kebele and religious leaders), HDA leaders, HEWs and health workers (district maternal and child health [MCH] officers, directors, health facility heads and health providers).

The sample size was determined through a process of data saturation when no new information or themes emerged from the additional data. After reaching 52 participants, the sample size was determined, ensuring enough participants for comprehensive and reliable results. The sample size was aimed at including a diverse group of participants from the study area, and data saturation was closely monitored throughout the data collection.

### Sampling methods

**FIGURE 2:**
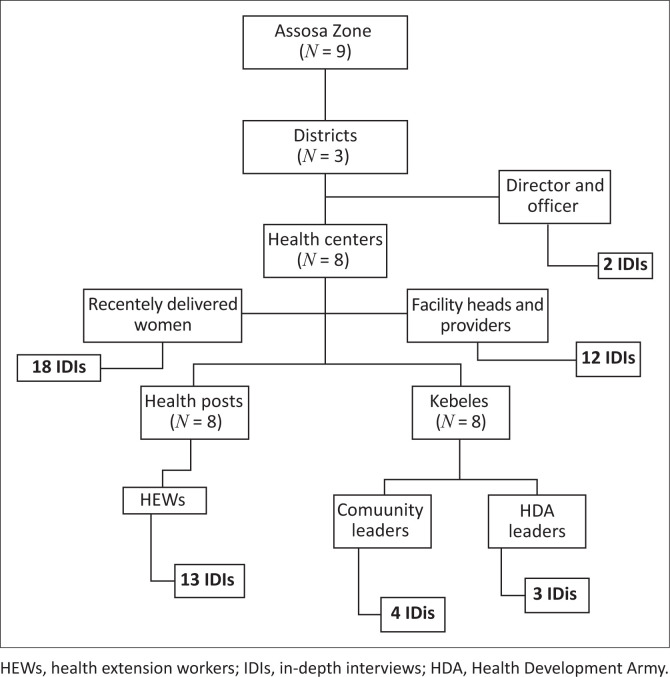
Sampling procedures for qualitative study.

Eight health centres from the districts were selected purposively for this study. Under each selected health centre, one health post and one Kebele was further selected based on the feasibility of the study. Lastly, study participants were sampled from each district health office, health centre and Kebele (Figure 2).

The study employed a purposive sampling method involving health workers and community leaders to select participants who met the inclusion criteria and represented diverse perspectives that ensured a comprehensive representation of various experiences. The participants were chosen based on their previous MNH CoC service utilisation experiences from recognised health facilities, knowledge and contributions about MNH CoC services provision at community and health facility levels (Figure 2). Data collectors contacted study participants to clarify the study objectives and the significance of their responses to the study. Participants were assured of the confidentiality of their responses and those who expressed interest in participating provided verbal consent. Participants had the opportunity to ask questions, and those who agreed to participate voluntarily proceeded with the IDIs.

### Data collection method(s) procedure and collection tools

The study utilised a semi-structured, IDI guide to collect data. The IDI guide was developed in English after reviewing literatures^19,20,21,22,23^ and aligning it with the study’s objectives. The guide was translated into Amharic for clear communication and piloted with a small group to improve question clarity and flow.

The interview guide was utilised to maintain consistency throughout the data collection process. Participants were provided with comprehensive study information, allowing them to express their opinions in their preferred language, and were asked to consent if they chose to participate. Three experienced health workers conducted interviews, sharing tasks of interviewing, note-taking and recording participant responses. The interviews were conducted at the household level for Kebele leaders, religious leaders and HDA leaders. The interviews of women who gave birth in the past 9 months were conducted after exiting measles vaccination services for their children. The interviews of HEWs, district MCH officers, directors, health facility heads and health providers were also conducted at their offices. The interviews were overseen by the Principal Investigator.

The primary question for the IDI was, ‘What are the barriers that influence non-use of MNH CoC (from ANC to PNC) services?’ Follow-up questions for further probing were transportation constraints, perceptions, religious influences, awareness and knowledge gaps, cultural influences and previous experiences of use of MNH CoC services. The IDI sessions took, on average, 25–55 min for each of the participants.

### Data management and analysis

The study involved bilingual transcriber’s transcribed audio recordings in participants’ native languages, then translated into the study language to preserve context and ensure comprehensive understanding. The Principal Investigator meticulously reviewed and listened to the recordings for accuracy. The Principal Investigator manually typed the transcripts of the individual interviews for analysis. Colaizzi’s^28^ seven steps of data analysis were used as a guide in the thematic analysis as follows:

1. The contents of the transcripts were read and re-read repeatedly to make sense of the IDIs.
2. Significant statements, phrases and sentences were extracted.
3. Pertinent statements or quotes with similar meanings were categorised into themes.
4. Steps 1–3 were repeated until all themes were generated and sub-themes were categorised.
5. A full and inclusive description was performed for all themes and sub-themes identified.
6. The report was generated by condensing the descriptions and the statements of the participants.
7. Verifying the findings.

The themes were created to respond to the qualitative objectives and were described in detail. To strengthen the descriptions and arguments, direct quotations were used. The identities of participants were disguised through pseudonyms as they narrated responses.

The study aimed to ensure the reliability and validity of its findings by establishing qualitative research criteria of credibility, transferability and conformability. These criteria were applied through audio recording of participant responses and the integration of the three data collector observations. The study ensured credibility by meticulously recording participants’ perceptions, integrating researcher observations and ensuring comprehensive data collection through repetitive questioning and participant member checking for accuracy and interpretation. Transferability was addressed by providing detailed descriptions of the research context of participants to ensure the applicability of findings to other situations. An audit trail was established to document all decisions made during the research process, demonstrating neutrality and objectivity. Triangulation of information through repetitive questioning ensured a comprehensive data collection process. The study ensured conformability by systematically analysing data, coding and categorising responses to accurately represent participants’ voices and maintain reliability in the findings. Direct quotes were used to authentically convey participants’ statements, ensuring the integrity and transparency of their contributions.

### Ethical considerations

Ethical clearance was obtained from the College of Human Sciences Research Ethics Review Committee of UNISA with NHREC registration number Rec-240816-05, CREC reference number 13112112_CREC_CHS_2021. Then, a support letter was written from the University of South Africa (UNISA) Ethiopian Centre to the BGR Health Bureau. The BGR Health Bureau wrote a support letter to the districts, and all districts also wrote support letters to all health facilities. Finally, informed consent was obtained from each participant before the data collection process commenced after the aim of the study was explained. Participants were also informed of their rights, including the right to withdraw at any time if they faced any discomfort during the data collection. The confidentiality of the information was also assured.

## Results

The study involved 52 participants, with 29% aged 25–34 years. About 29 and 22 participants were Muslims and Orthodox Tewahedo followers, respectively. Over half attended college and university, while 15 attended primary and secondary schools. The remaining (seven) had no formal education. Over half were employed, while 17 were farmers. The remaining participants were merchants, housewives or unemployed (see Table 1).

**TABLE 1:** Demographic characteristics of study participants in Assosa Zone, north-western Ethiopia (*N* = 52).

Variables	Category	Frequency
Type of participants	Women who gave birth in the past 9 months	18
	HEWs	13
	Midwives†	2
	Heads (MCH department heads, technical heads and facility heads)‡	10
	Experts	2
	Religious leader	1
	Kebele leaders	3
	Health development team leader	3
Age (years)	15–24	7
	25–34	29
	35–44	13
	≥ 45	7
Sex	Male	12
	Female	40
Religion	Muslim	29
	Orthodox	22
	Protestant	1
Education	No formal education	7
	Primary	11
	Secondary	4
	College and University	30
Occupation	Farmer	17
	Housewife	1
	Employee	30
	Merchant	2
	Unemployed	2

MCH, maternal and child health; HEWs, health extension workers.

† , Midwives (BSc).

‡ , Diploma midwives, BSc midwives, BSc nurses, Health officers (BSc) and Masters in Public Health Professionals.

Two main themes and 10 sub-themes that hindered the completion of MNH CoC services were identified. The barriers to women’s completion of MNH CoC services include economic constraints, a lack of knowledge, workload, previous experiences, delayed first ANC booking, perceived healthiness, fear of notifying pregnancy, fear of using PNC services, husband influence and religious perceptions (Table 2).

**TABLE 2:** Themes and sub-themes that influence the utilisation of maternal and neonatal health continuum of care services in Assosa Zone, north-western Ethiopia (*N* = 52).

Themes	Sub-themes
1. Individual barriers	1.1 Economic barriers for transportation and medication costs 1.2 Lack of knowledge or awareness 1.3 Workload of women 1.4 Previous experiences and perceived good health 1.5 Delayed 1st ANC start
2. Cultural and community-related barriers	2.1 Religious barriers, community perceptions and husbands’ influence 2.2 Fear of announcing pregnancy 2.3 Fear of using postnatal care

ANC, antenatal care.

### Theme 1: Individual barriers

#### Sub-theme 1.1: Economic barriers for transportation and medication costs

Most of the study participants stated that one’s economic status influences the completion of MNH CoC services. The study participants reported that even though MNH services were free, mothers experienced difficulties with increasing transportation costs. Many mothers further decried having to buy drugs from private pharmacies and clinics, which they could not afford:

‘To receive the CoC, I have to travel long distance with high cost. Health workers ordered me to buy medication from private clinics; hence, I discontinued CoC.’ (28 years old, female, farmer)

‘During my present pregnancy, I had a bleeding history and went to a health centre. After they provided anaemia medicine [*said iron folic acid*], they told me that my problem was beyond their capacity and ordered me to go to higher facilities. I went to a private clinic, and they advised, counselled, and gave me another better anaemia medicine [*said coated iron folic acid*] and injection. My bleeding was stopped, and I followed about 5–6 times after my first visit. I did not want to tell you the cost, I lost, large amount of money. I got these services with high cost. I did not know what happened if this occurred to the poor.’ (40 years old, female, merchant)

Women opposed referral even for serious complications because of transportation and other non-service-related expenditures. A midwife who was a service provider expressed it as follows:

‘If the cases need referral and we consulted to refer them to higher facilities, they resisted the referral and said that they did not have any thing [*transportation, close for neonates and money for food*] and they preferred to get services in this facility. In the future, I fear that women decline to utilise CoC services due to the economic burden.’ (28 years old, female, midwife)

The Ethiopian government availed ambulance services for each health facility for the management of emergencies and delivery services. However, many women, especially from rural areas, complained that they could not receive ambulance services and discontinued MNH CoC services because of economic barriers to transportation:

‘The ambulance was bought through our community shares, but our mother, sisters, and wives were not served well. Last year three women and this year one woman gave birth at home in our Kebele because of transportation problems.’ (52 years old, male, community leader)

Supporting the above, a health extension worker elaborated:

‘Delivery in health posts is not considered as skilled delivery. However, some women preferred to deliver at health posts due to feared transport costs.’ (37 years old, female, HEW)

A district MCH director who leads Maternal, Neonatal and Child Health services supported the ideas of the community leader and HEW as follows:

‘Some women did not attend CoC services due to lack of transportation costs, especially distant Kebele residents. During delivery, all women did not get ambulance services because of shortage of ambulance and shortage of fuel.’ (35 years old, male, MCH director)

#### Sub-theme 1.2: Lack of knowledge or awareness

Poor awareness contributed to a lack of skilled MNH CoC services. One of the community leaders explained that women did not attend ANC services because of awareness problems in their Kebele’s as follows:

‘Women were not conducted ANC attendance from a skilled provider due to awareness gaps.’ (52 years old, male, community leader)

All women could not attend recommended MNH CoC services because of awareness gaps on the advantages of facility CoC services, as stated by one of the study participants at the community level:

‘In our Kebele, majority women know the advantage of facility CoC services. However, some women did not know it. Due to this, they did not receive all recommended MNH CoC follow-ups.’ (32 years old, female, HEW)

The study identified that along the CoC, women’s lack of awareness or knowledge contributed to their dropout or incomplete utilisation of CoC services. There was a lack of awareness and the presence of knowledge gaps that influenced the completion of the MNH CoC, as described by one of the women as follows:

‘I started ANC within three months of pregnancy and conducted three times after first ANC follow-ups. I delivered my child at a health facility. I did not receive PNC services from the health centre because of lack of knowledge to receive it.’ (22 years old, female, farmer)

Poor awareness about complications among women contributed to a lack of skilled maternal health services, as they could not understand the risks associated with complications. One of the recently delivered women described it as below:

‘… [*T*]hen I did not conduct PNC services because health workers [*had*] not appointed me to return for PNC services and I did not have awareness to conduct it at health centres. After 37 days, I developed vaginal bleeding.’ (28 years old, female, farmer)

#### Sub-theme 1.3: Workload of women

The workload in the household is one of the barriers that hinder women from accessing MNH CoC services. The workload affected the effective use of the continuum of MNH services as the HEW described below:

‘In our Kebele, majority women utilise MNH services. However, there are barriers to hinder them to utilise the CoC services. Some women were busy at the household level, and they could not start ANC within four months of their pregnancy.’ (32 years old, female, HEW)

#### Sub-theme 1.4: Previous experiences and perceived good health

Low perceptions of risk along the CoC contributed to women not seeking MNH services if they felt well during their postnatal periods. Women commented that they used maternal health services, especially PNC services when they felt they had maternal and neonatal complications after delivery. The participants in this study confirmed that they did not seek care unless they had complications. One participant described this as follows:

‘I started ANC at four months of pregnancy, conducted four consecutive ANC follow-ups, delivered at a health centre and waited for about 24 hours. After delivery, I could not attend PNC because I and my neonate were healthy. If I were sick, I would go to a health centre.’ (35 years old, female, farmer)

‘I started first ANC at five months, completed all ANC services, delivered at the health centre, and waited at 24 hours at the delivered health facility. However, I did not go to health facilities to attend other PNC services because I felt healthy. I think someone went to the health facility when there are danger signs like bleeding.’ (32 years old, female, employed)

The absence of any complications in previous pregnancies gave women a low perception of their risk in the current pregnancy. These experiences contributed to dropouts from the MNH CoC services among many women. One of the health centre heads expressed the effect of the lack of complications for the completion of MNH CoC services as follows:

‘Those women who had no previous health complications related to pregnancy, delivery, and postnatal periods, they perceived that their recent pregnancy was similar to the previous and they did not utilise the CoC services from health facilities.’ (42 years old, male, health center head)

#### Sub-theme 1.5: Delayed 1st ANC start

The completion of MNH CoC services is significantly influenced by the late start of ANC follow-up services from skilled providers. However, many women delayed the initiation of the first ANC services, resulting in incomplete utilisation of MNH CoC service. One of the service users stated:

‘I started ANC at the fifth month of pregnancy and conducted up to three follow-ups. I did not utilise the fourth ANC follow up because of delayed first ANC.’ (30 years old, female, farmer)

The main reasons that caused the delayed initiation of the first ANC were the absence of previous complications, more pregnancies and unwanted pregnancies. These resulted in incomplete utilisation of MNH CoC services. One of the participants explained the main reasons as follows:

‘Those women who were primigravida and had hyperemesis were started ANC within shorter months of pregnancy. However, those women who delivered three and more and had no history of complications in their previous pregnancy and delivery were delayed starting ANC services. Some women were hidden due to short intrapartum [*unwanted*] pregnancy. They feared their neighbours and HEWs. They started when their pregnancy was visible, and they did not receive all required MNH CoC services.’ (30 years old, female, midwife)

The delay in commencing the first ANC visit reportedly occurred at times because of irregularity of women’s menstrual period after using family planning. One of the participants explained the effect of this barrier as follows:

‘Health Development Army identified pregnant women from 2–3 months of their pregnancy. They referred to the health post and we [*HEWs*] referred to the health centres for ANC follow-ups. In most of the time, women delayed starting ANC visits. The dalliance occurred for those women who used family planning lacks regularity of their menstrual periods. After removal, their menstrual period was delayed as in the previous. They delayed notifying their pregnancy up to 5–6 months of pregnancy.’ (26 years old, female, HEW)

### Theme 2: Cultural and community-related barriers

#### Sub-theme 2.1: Religious barriers, community perceptions and husbands’ influence

Religion was identified by most of the study participants as the most determining barrier to the continuity of MNH services. One participant stated:

‘In rare conditions, a few women [*Muslim followers*] did not volunteer to receive CoC services from male health workers. They did not allow seeing their bodies with other males other than their husband which is linked to religious thoughts.’ (35 years old, male, MCH director)

Another participant explained:

‘During labour and delivery, women did not prefer to be delivered by males. They rose that their religion was not allowed to touch females by males other than their husbands.’ (26 years old, female, HEW)

Women’s misperceptions were factors that deterred them from utilising the components of postnatal services. Women believed that family planning decreased the quality of breast milk and resulted in thinness, as described by one of the community HEWs as follows:

‘The main barriers in our Kebele that hinder [*women*] to utilise MNH CoC were the presence of perceptions. Some women believed that family planning causes thinness of women and minimise the quality of breast milk. They refused to take IPPFP after delivery.’ (37 years old, female, HEW)

Husbands’ influences were barriers to the utilisation of components of MNH CoC services, such as negative attitude to immediate post-partum family planning (IPPFP), as identified by many participants. One participant explained as follows:

‘The main barrier for our Kebeles that hinder to utilise IPPFP was the influences of husbands. Husbands believed that family planning services [*were*] not acceptable in respect of their religion. Some husbands need more children and women feared divorce if they took family planning.’ (37 years old, female, HEW)

#### Sub-theme 2.2: Fear of announcing pregnancy

Many study participants expressed fear of revealing their pregnancy to the community, fearing judgement or criticism, and worried about the impact on their relationships and social standing. This practice affected early ANC initiation and frequent follow-ups. One of the HDA leaders explained as follows:

‘Some women did not notify their pregnancy at early times and delayed their first ANC follow-ups until 5–6 months.’ (50 years old, female, HDA leader)

‘As culture of Benishangul [*Bera*] community, when some women became pregnant, they did not want to tell their pregnancy early or within 16 weeks of pregnancy. Following this, the women did not receive fourth ANC follow-ups. Some women hide their pregnancy up to 30 weeks and some women became delivered without any ANC follow-ups.’ (35 years old, male, MCH director)

Women alluded to non-disclosure of pregnancy in its early stages. Closely spaced pregnancies were often viewed as shameful, signifying irresponsibility or lack of planning, leading to social stigma and judgement by the communities as they were often shamed. Pregnancy is concealed until it becomes conspicuous to others, as stated by one of the participants as follows:

‘Few women hid their pregnancy until it was visible, especially those women who were pregnant within less than three years of following their previous pregnancy. They considered that it is a shame to notify their closed spaced pregnancy to health workers, community and HEWs.’ (41 years old, male, community leader)

Multiparous women with husbands controlling family planning services often hide pregnancies from community, fearing retaliation or abuse. This leads to unsafe pregnancies, inadequate prenatal care and complications for both mother and baby, compromising women’s reproductive autonomy and health:

‘In most of the time, those who had many children hidden their early pregnancies due to fear of their neighbours, culture and economic influences. Women experienced male influenced for the utilisation of family planning services, declined to notify their pregnancies to women development army leaders.’ (36 years old, male, MCH officer)

Cultural influences such as falling pregnant while unmarried were a hindrance to the uptake of MNH CoC services. Unmarried women concealed their pregnancy because this was construed as a disgrace in their community:

‘Women who had husbands, disclosed their pregnancies, but those who did not have husbands hid their pregnancies.’ (27 years old, female, HEW)

To some extent, women believed that a fetus needed to be large before announcing a pregnancy. The reasons for non-timely initiation of ANC services or public revelation of early pregnancy were feelings of embarrassment in the belief that it is presumptuous to talk of pregnancy when miscarriage cannot be ruled out. One participant said:

‘Some women feared early disclosure of pregnancy. They think that the fetus needs to be large to notify to others. Otherwise, people may think that there were miscarriages.’ (42 years old, male, health centre head)

#### Sub-theme 2.3: Fear of using postnatal care

There were traditional beliefs (perceptions) and religious practices that hindered the completion of MNH CoC services among women. Generally, women did not attend PNC services because of the community’s traditional beliefs. Women feared that an imbalance in the air would make them and their neonates ill, and they believed that they should stay home after giving birth. Such beliefs influenced women not to attend PNC services:

‘The community believed that women were better to stay home after delivery for up to 40 days. They [*community*] believed that the body of the woman [*was*] not strong enough and she will [*become*] sick due to air imbalance. This [*prevents*] women not to receive PNC services unless they have any sickness.’ (41 years old, male, community leader)

‘During PNC, we have been conducted from home to home. Because women feared that they became ill due to the imbalance of air.’ (30 years old, female, HEW)

‘… [*W*]hen they were at the health centre, they feared more the light of the sun.’ (29 years old, female, midwife)

## Discussion

The two key themes and 10 sub-themes emerged from the study participants that hinder the completion of MNH CoC service utilisation in Assosa Zone, north-western Ethiopia. The barriers to women’s completion of MNH CoC services were economic constraints, lack of knowledge, women’s workloads, previous experiences, delayed first ANC booking, perceived healthiness, fear of notifying pregnancy, fear of using PNC services, and husband influence and religious perceptions. These themes and sub-themes provide insights into areas needing attention and improvements to ensure high-impact MNH CoC services.

The study found that economic barriers, such as transportation and medication costs, hinder the completion of MNH CoC services. Despite MNH services being free, mothers faced difficulties with rising transportation costs, leading them to criticise purchasing drugs from private pharmacies and clinics because of their inability to afford these expenses. Economic barriers because of transportation and medication costs were the most reported barriers to obtaining comprehensive MNH CoC services.^29,30^ A study in Ethiopia found that women faced limited access to lifesaving services because of low socio-economic status and non-utilisation being concentrated among the poorest.^31^ Other evidence proved that direct and indirect costs were significant barriers that hindered MNH care utilisation.^32^ Studies in Nepal and sub-Saharan Africa have also shown that the completion of MNH CoC services is influenced by higher health services-related costs.^33,34^

In the Ethiopian context, to reduce maternal and neonatal deaths, all pregnant women are encouraged to utilise ambulance services to give birth in health facilities. According to Ministry of Health guidelines, ambulances should link rural health posts to health centres and health centres with hospitals through a referral system if women need emergency, maternal, obstetric and neonatal care.^35^ However, in this study, most participants complained that many women were not utilising CoC services because of a lack of emergency transportation. The present study also identified that women delivered at home because of a lack of emergency transport services. This happened because of the shortage of fuel and shortage of ambulances. A similar study finding revealed that ambulance utilisation among women was low because of a ratio that reflected that there was a low number of ambulances to women in the area, shortage of fuel, maintenance problems and unavailability of drivers.^36^

The workload of women was one of the barriers that hindered women from accessing MNH CoC services. Women have a responsibility for the household chores, taking care of children and supporting their husbands in the agricultural fields during the harvest. A similar study in South Sudan has shown that women with high household workloads were less likely to complete the fourth ANC visit because of the late start of the first ANC visits.^37^ Studies conducted in Ethiopia and South Sudan reported that women were unable to attend ANC delivery and PNC services when there was no family support to take care of other children.^32,37^ In middle-income countries, women are responsible for household care, childcare and supporting husbands during harvest.^37^ A study in Gambia also supported this finding, with women often working long hours and not attending institutional healthcare services.^38^

Findings in this study have shown that women’s lack of awareness about the importance of MNH CoC services and the risks associated with complications leads to delayed ANC follow-ups or discontinuation of these services. This is consistent with the findings of the studies conducted in Somalia, Kenya and Ethiopia, where poor awareness and information about reproductive health contributed to a lack of skilled utilisation of MNH CoC services.^20,21,39^ Additionally, lack of menstrual knowledge and irregularity of menstruation periods after family planning led to delayed ANC follow-ups, as supported by a Kenya study.^39^ Many study participants confirmed that women did not seek care unless they had complications during pregnancy or after delivery. The absence of complications or low perception of risk of women’s previous pregnancy hindered women from using MNH CoC services because they expected their present pregnancy to be like the previous one. On the other hand, previous complications contributed to the use of routine MNH CoC services, while women who had safe home deliveries dropped the CoC services. The perceptions were mostly because of a lack of knowledge and understanding of the benefits of MNH care services, which led to not seeking care until they recognised their illness. This indicates that MNH services in the study area were an incomplete utilisation of CoC services among mothers. The findings were consistent with other studies, where the utilisations of CoC were influenced by intra- and postpartum complications, individual experiences and perceptions of the necessity of PNC when obstetric complications were recognised.^21,40^ Different studies have shown that low levels of awareness and perceived absence of health problems were the identified gaps that caused the discontinuation of MNH CoC services from skilled providers.^21,23,40^ Antenatal care attendance was not viewed as a routine exercise but was linked to pregnancy complications. Women also perceived PNC services as necessary only if there were obstetric complications, and thus, they always stayed home until they became ill.^20^

Early attendance of ANC is crucial for improving MNH outcomes.^41^ While late initiation of ANC leads to service discontinuation across the CoC pathways. The first ANC should be initiated before 16 weeks of gestational age, as it aids in the early detection of pregnancy-related complications.^42^ In this study, factors such as the absence of complications, unintended pregnancy, unknown pregnancy after family planning and previous pregnancy complications also affected early ANC initiation. Women with more previous births or pregnancies were found to be delaying initiation of the first ANC booking. Unintended pregnancy can also negatively affect early ANC initiation, as the woman may not want to disclose her secretively kept pregnancy to neighbours and health workers. This is in line with findings in an earlier study where women who started ANC in later trimesters did not receive recommended ANC visits, resulting in the discontinuation of MNH services across the continuum pathways.^40^ This early initiation of ANC is critical to aid in the early detection of pregnancy-related complications, including low birth weight, stillbirth, intrauterine and foetal death.^42^ These findings were also supported by a systematic and meta-analysis study where women with pregnancy complications were more likely to start early ANC than those without complications, but those without previous issues could not attend ANC services.^43,44^ According to a study conducted in Jimma, Ethiopia, pregnant mothers who had more children were more likely to delay than those who had fewer children.^45^ Unintended pregnancy is another barrier to the early initiation of the first ANC booking because the mother does not want to expose her secrets to neighbours and health workers.^44,46^ Pregnancy with an unknown last menstrual period results in the late initiation of ANC follow-ups, especially in women who use family planning, which results in an absence of menstrual periods, and this might deter women from starting their ANC early.^40^

The present study showed that gender preference was an important barrier for women to access MNH CoC services because of cultural and religious obligations. Some Muslim-follower women were uncomfortable showing their body parts to male health workers because they believed that the only person seeing their bodies is their husbands. This was supported by a study conducted in Kenya where women were uncomfortable with male providers in conformity with the Islamic faith and their culture.^47^ Another study conducted in Nepal found that women do not want to visit hospitals because they are uncomfortable showing their body parts to male doctors.^48^ The present study is also supported by an Ethiopian study where community misperceptions, cultural restrictions and negative attitudes towards male midwives linked to religious faith among Muslim women were the most significant barriers to accessing CoC services.^40^

Religious prohibition and husbands’ influence were barriers for women in family planning utilisation in the present study. In line with the study findings, studies conducted in Liberia and Ethiopia identified fear of side effects, partner opposition and the presence of religious restrictions as the major barriers that hinder women’s utilisation of IPPFP services.^49,50^

Women’s misperceptions were determinant factors that impeded them from utilising the components of postnatal services such as IPPFP because of the presence of perceptions that family planning decreased the quality of breast milk and caused female thinness. This was supported by a study conducted in Kenya where women believed that family planning reduced the production of milk, and they did not utilise family planning methods because of fear of side effects.^51^ Like the present finding, studies conducted in Ethiopia indicate that fear of side effects is one of the barriers to the utilisation of IPPFP among women.^52^

The tradition of early pregnancy secrecy has hindered women from utilising all recommended ANC services because of fear of judgement or discrimination, resulting in missed opportunities for vital health screenings and interventions that could improve mother and baby outcomes. This agreed with other studies conducted in Ethiopia, Malawi and Ghana where the tradition of concealing pregnancy in early stages affects ANC follow-ups.^21,47,53^ Incompletion of all recommended ANC service attendance among pregnant women is common in Ethiopia.^43,44,45^ Like previous studies, the traditional concealment of pregnancy prevents women from completing ANC because study participants believed that people should only know after the baby was born or when the abdomen grew large, as it was shameful to announce the pregnancy in case a miscarriage occurred.

In this study, there were traditional beliefs (perceptions) and religious practices that hampered the completion of MNH CoC services among women. The community believed that it was better for women to wait at home after delivery for up to 40 days. They believed that their bodies were not yet strong enough and hence, they could become sick because of air imbalance. This belief prevented women from receiving PNC services. Similar evidence has shown that recent births have been delayed by 45 days because of potential risks to the mother and neonates, who are believed to be vulnerable to Satan’s evil. Women might face serious health issues, such as bleeding profusely, and may even die if they go outside their homes. They are also forbidden to leave the house or touch anyone for 11 days after delivery.^48,54,55^

### Strengths and limitations

The study aimed to capture the topic of MNC CoC in the Assosa Zone, ensuring data saturation and allowing for meaningful conclusions. After conducting interviews with a diverse range of participants, no new information or themes emerged, indicating data saturation. The selection of participants was a strength, providing diverse perspectives on individual and cultural barriers. Direct interaction with health workers, HEWs, HDA leaders, Kebele leaders, religious leaders and women enriched understanding of barriers hindering completion of MNH CoC services. The study’s detailed descriptions of health provision at community and health facility levels and participant selection process bolstered the transferability of findings, offering a thorough account of individual and cultural barriers in the Assosa Zone. Facilitating comparisons with different viewpoints of the study participants with others is also another strength.

The study primarily focussed on barriers to MNH CoC services completion, neglecting contributing factors. This limited the understanding of diverse perspectives and the study’s scope and generalisability. The logistical constraints faced in accessing very distant Kebeles’ participants in Assosa Zone may have limited the representation of diverse perspectives. The focus on participants’ perceptions may not fully represent the whole viewpoint in the area and may not be applicable to diverse environments.

### Recommendations

To improve access to essential MNH services for poor women, income-generating interventions should be designed to reduce transportation and medication costs. This can be achieved through training for small businesses or financial assistance for transportation and healthcare expenses. Health insurance systems should also be introduced to alleviate the financial burden on women seeking MNH CoC services, ensuring they receive necessary care without facing financial hardship.

District and facility leaders should enhance accountability in ambulance administration to improve MNH CoC services. This can be achieved by implementing clear protocols, monitoring systems and regular training for ambulance drivers. This will ensure timely and efficient response to MNH emergencies, especially during labour and delivery, thereby improving the quality of care provided during emergencies.

Women should actively participate in pregnant women conferences and women’s HDA network systems to gain awareness about skilled attendance of ANC, childbirth and PNC services. Regular awareness-raising interventions, such as home visits, health education and arranged meetings, should be conducted by health development leaders and HEWs. Community, Kebele and religious leaders should collaborate with HDA Networks, HEWs and health workers to create awareness and reinforce service provision by male health workers, especially Muslims. Open communication with husbands can help break down barriers to MNH utilisation.

## Conclusions

The article established that economic circumstances served as barriers to completing lifesaving MNH CoC services for women. Such barriers manifest themselves in access to appropriate transport to receive MNH CoC services and unaffordable medication. The study found that early booking of ANC services, perceived low risk of complications, history of uncomplicated pregnancy and delivery with previous pregnancies, awareness gap of MNH CoC services, presence of workload in households, secreting pregnancy, traditional beliefs to stay at home during PNC period, husband influence and religious influences all accounted as barriers to the completion of MHN CoC services in the study area.
